# Supplementary material for: Carriage of antimicrobial-resistant Enterobacterales among pregnant women and newborns in Amhara, Ethiopia
Source: Int J Infect Dis. 2024 Jun;143:None. doi: 10.1016/j.ijid.2024.107035 (PMC11068590; doi:10.1016/j.ijid.2024.107035)
Supplement: Supplementary file 1 [file mmc1.docx]

**Carriage of antimicrobial-resistant Enterobacterales among pregnant women and newborns in Amhara, Ethiopia**

# Supplementary Material

## Contents

**Supplementary Methods.** Detailed sample processing methods for identification and antimicrobial susceptibility testing of specimens.

**Supplementary Figure 1.** 2x2 tables comparing carriage of ESBL-producing organisms, CRE, and GBS in mothers during labor/delivery and their neonates.

**Supplementary Table 1.** Primer and probe sequences for *bla_-_*_TEM_*, bla_-_*_SHV_ and *bla_-_*_CTXM_ gene detection.

**Supplementary Table 2.** PCR results among 101 ESBL-producing isolates, as identified by AST. PCR was not conducted for 1 isolate.

**Supplementary Table 3.** Antimicrobial resistance genes detected among 16 CRE.

### **Supplementary Methods.** Detailed sample processing methods for identification and antimicrobial susceptibility testing of specimens.

## Antimicrobial susceptibility testing

Phenotypic identification and determination of antimicrobial susceptibility were performed at NICD, a division of National Health Laboratory Service (NHLS), South Africa. Interpretation of susceptibility breakpoints was according to Standard Clinical and Laboratory Standards Institute (CLSI) guidelines [[1]](https://www.zotero.org/google-docs/?XNpj4E).

Samples received at NICD were processed upon receipt in the laboratory. A worksheet was created for each batch of processed swabs. Each swab was plated on following media: (1) MacConkey agar with an imipenem disc placed on the initial inoculum as an additional screen for carbapenem resistant organisms, (2) Colorex™ESBL for detection of Gram-negative bacteria producing Extended Spectrum Beta-Lactamase (ESBL), (3) Colorex™mSuperCARBA™ for detection and isolation of Carbapenem-Resistant *Enterobacterales* (CRE), and (4) Todd Hewitt enrichment broth for *Streptococcus agalactiae* (GBS). Plates and broths were incubated for 24-hours at 37^o^C.

Colorex™ agar plates were examined for colonies demonstrating various color changes according to the manufacturer's instructions for each of the Colorex™ media used. MacConkey agar plates were examined for Gram negative bacteria growing close to the imipenem disc. Suspected colonies on Colorex™ media were plated out on MacConkey agar for identification and antimicrobial susceptibility testing. After 24-hours of incubation, colonies were identified using the MALDI TOF-MS Biotyper® system (Bruker Daltonics GmbH, Bremen, Germany) and AST performed on MicroScan® (Beckman Coulter, Inc., West Sacramento, CA, USA) using Microscan® Neg MIC Panel Type 44. The Todd Hewitt enrichment broth was sub-cultured on a blood agar plate and Colorex™StrepB agar plate and incubated for 24 hours at 37^o^C.

Suspected colonies from blood agar were identified on the MALDI Biotyper®. Suspected colonies from Colorex™StrepB agar were plated out on blood agar prior to identification and AST. Isolates of GBS underwent MIC testing using the Sensititre (ThermoFisher). *Streptococcus* species STP6F Trek panel as well as Kirby Bauer disc susceptibility testing. Isolates of ESBL-producing organisms, CRE and GBS, were stored in tryptic soy broth with 10% glycerol in -70^o^C.

## Molecular testing

Molecular testing was also conducted to verify the presence of ESBL genes among isolates identified as ESBL-positive by antimicrobial susceptibility testing profiles to the third and fourth generation of cephalosporins identified by MicroScan®. The DNA was extracted using a crude boiling method at 95˚C for 25 minutes. The DNA templates were then tested for the presence of extended-spectrum beta-lactamase (ESBL) genes such as *bla_-_*_TEM_*, bla_-_*_SHV_ and *bla_-_*_CTXM_ using the LightCycler 480 instrument (Roche Applied Science, Germany) and LightCycler 480 Probes Master kit (Roche Diagnostics, USA) in a real-time polymerase chain reaction (PCR) assay. The *bla_-_*_TEM_*, bla_-_*_SHV_ and *bla_-_*_CTXM_ genes were amplified by multiplex real-time PCR using the primers and probes shown in **Supplementary Table 1**. For *bla_-_*_TEM and_ *bla_-_*_SHV,_ the reaction conditions were 10μM primers, 2μM probes, denaturation for 95°C for 5 minutes, and then 45 cycles of 95°C for 10 seconds, 55°C for 30 seconds and 72°C for 1 second. The *bla_-_*_CTXM_ PCR was a multiplex assay targeting *bla_-_*_CTXM_ group M1 and M2-9 using primer and probe sequences as described previously [[2]](https://www.zotero.org/google-docs/?TCG9aI).

##

## Whole genome sequencing

Whole genome sequencing was conducted to verify the presence of CRE genes among isolates identified as CRE-positive by antimicrobial susceptibility testing profiles to carbapenems identified by MicroScan®. Genomic DNA (gDNA) was extracted using QIAamp DNA mini kit (Qiagen, TX, USA) following the manufacturer’s instructions. The concentrations of the extracted gDNA were determined using Qubit 4.0 fluorometer (ThermoFisher Scientific, Waltham, MA, USA). Multiplexed paired-end libraries were prepared using the Illumina DNA Prep kit (Illumina, San Diego, CA, USA). Sequencing was performed on Illumina NextSeq 550 platform (Illumina, San Diego, CA, USA) (2x 150bp) with 100x coverage at the NICD Sequencing Core Facility, NHLS, South Africa.

Raw paired-end reads were analysed using the Jekesa pipeline (v1.0; https://github.com/stanikae/jekesa). Briefly, Trim Galore! (v0.6.2; https://github.com/FelixKrueger/TrimGalore) was used to filter the generated sequence raw reads (Q >30 and length >50 bp). De novo assembly and optimization of the contigs were performed using SPAdes v3.13 and Shovill (v1.1.0; https://github.com/tseemann/shovill), respectively. The multilocus sequence typing (MLST) profiles were determined using the MLST tool (version 2.16.4; https://github.com/tseemann/mlst). Assembly metrics were calculated using QUAST (v5.0.2; <http://quast.sourceforge.net/quast>).

Whole-genome single nucleotide polymorphism (SNP) differences were determined with a reference-free approach using the SKA toolkit [[3]](https://www.zotero.org/google-docs/?J8EeHR). Antibiotic resistance profiles and virulence genes were predicted using ABRicate (version 1.0.1; https://github.com/tseemann/ABRicate), against the Comprehensive Antibiotic Resistance Database (CARD), ResFinder - Center for Genomic Epidemiology (CGE) database [[4]](https://www.zotero.org/google-docs/?IlGP3L), NCBI AMRFinderPlus [[5]](https://www.zotero.org/google-docs/?nohvxY), and Virulence Factor Database (VFDB) [[6]](https://www.zotero.org/google-docs/?fIyBVF) implemented in the Jekesa pipeline. Pathogen Watch (https://pathogen.watch/) was used to construct the phylogenetic tree [Newick (NWK) file]. The exported NWK file was used in Microreact (https://microreact.org/showcase) to visualize and edit the phylogenetic tree. The assembled genome files were submitted to the National Center for Biotechnology Information GenBank and are available under BioProject number: PRJNA819852[RM1].

**References**

[[1] CLSI M100 : Performance Standards for Antimicrobial Susceptibility Testing. 2022.](https://www.zotero.org/google-docs/?JE57AT)

[[2] Birkett CI, Ludlam HA, Woodford N, Brown DFJ, Brown NM, Roberts MTM, et al. Real-time TaqMan PCR for rapid detection and typing of genes encoding CTX-M extended-spectrum beta-lactamases. J Med Microbiol 2007;56:52–5. https://doi.org/10.1099/jmm.0.46909-0.](https://www.zotero.org/google-docs/?JE57AT)

[[3] Harris SR. SKA: Split Kmer Analysis Toolkit for Bacterial Genomic Epidemiology. Genomics; 2018. https://doi.org/10.1101/453142.](https://www.zotero.org/google-docs/?JE57AT)

[[4] Bortolaia V, Kaas RS, Ruppe E, Roberts MC, Schwarz S, Cattoir V, et al. ResFinder 4.0 for predictions of phenotypes from genotypes. J Antimicrob Chemother 2020;75:3491–500. https://doi.org/10.1093/jac/dkaa345.](https://www.zotero.org/google-docs/?JE57AT)

[[5] Feldgarden M, Brover V, Haft DH, Prasad AB, Slotta DJ, Tolstoy I, et al. Validating the AMRFinder Tool and Resistance Gene Database by Using Antimicrobial Resistance Genotype-Phenotype Correlations in a Collection of Isolates. Antimicrob Agents Chemother 2019;63:e00483-19. https://doi.org/10.1128/AAC.00483-19.](https://www.zotero.org/google-docs/?JE57AT)

[[6] Joensen KG, Scheutz F, Lund O, Hasman H, Kaas RS, Nielsen EM, et al. Real-time whole-genome sequencing for routine typing, surveillance, and outbreak detection of verotoxigenic Escherichia coli. J Clin Microbiol 2014;52:1501–10. https://doi.org/10.1128/JCM.03617-13.](https://www.zotero.org/google-docs/?JE57AT)

###

### **Supplementary Figure 1.** 2x2 tables comparing carriage of ESBL-producing organisms (a), CRE (b), and GBS (c) in mothers during labor/delivery and their neonates.


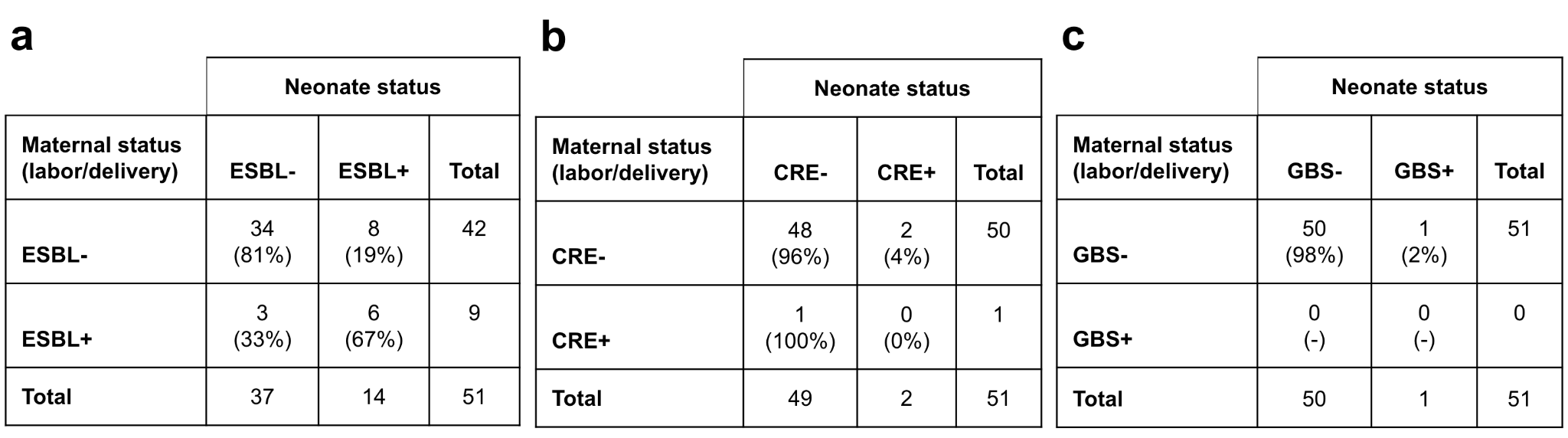


### **Supplementary Table 1.** Primer and probe sequences for *bla_-_*_TEM_*, bla_-_*_SHV_ and *bla_-_*_CTXM_ gene detection.

| **ESBL primer and probe name** | **Primer and probe sequences** |
| --- | --- |
| TEM Forward Primer | 5’- AAG TTC TGC TAT GTG GTG CGG TA -3’ |
| TEM Reverse Primer | 5’- TGT TAT CAC TCA TGG TTA TGG CAG C -3’ |
| TEM A Primer | 5’- GTA AGA TGC TTT TCT GTG ACT GGT GA -3’ |
| TEM S Primer | 5’- AGT TCT GCT ATG TGG TGC GGT ATT A -3’ |
| TEM Probe | 5’- FAM- TGC GGC GAC CGA GTT GCT CTT –BBQ -3’ |
| SHV Forward Primer | 5’- CAG CAG GAT CTG GTG GAC TAC T -3’ |
| SHV Reverse Primer | 5’- GTC AAG GCG GGT GAC GTT -3’ |
| SHV A Primer | 5’- AAG GCG GGT GAC GTT GTC -3’ |
| SHV S Primer | 5’- CCG GTC AGC GAA AAA CAC -3’ |
| SHV Probe | 5’- Cy5- TCT GGC GCA AAA AGG CAG TCA –BBQ -3’ |
| CTX-M Forward Primer | 5’- ATG TGC AGY ACC AGT AAR GTK ATG GC -3’ |
| CTX-M Reverse Primer | 5’- ATC ACK CGG RTC GCC NGG RAT -3’ |
| CTX-M1 Probe | 5’- FAM- CCC GAC AGC TGG GAG ACG AAA CGT -BBQ -3’ |
| CTX-M2 Probe | 5’- YAK- CAG GTG CTT ATC GCT CTC GCT CTG TT -Q -3’ |
| CTX-M9 Probe | 5’- 610- CTG GAT CGC ACT GAA CCT ACG CTG A –Q - 3’ |
| CTX-M9all/1 Probe (locked nucleic acid [LNA] Probe) | 5’- 640- CG+AC+AAT+ACN GCC+ATG+AA –BBQ -3’ |

### **Supplementary Table 2.** PCR results among 101 ESBL-producing isolates, as identified by AST. PCR was not conducted for 1 isolate.

| **Gene** | **Count (%)** |
| --- | --- |
| TEM only | 3 (3.0) |
| SHV only | 1 (1.0) |
| CTX-M-1 only | 31 (30.7) |
| CTX-M-9all only | 2 (2.0) |
| TEM + SHV | 1 (1.0) |
| TEM + CTX-M-1 | 43 (42.6) |
| TEM + CTX-M-9all | 4 (4.0) |
| SHV + CTX-M-1 | 6 (5.9) |
| CTX-M1 + CTX-M-9all | 1 (1.0) |
| TEM + SHV + CTX-M-1 | 6 (5.9) |
| None | 3 (3.0) |

###

### **Supplementary Table 3.** Antimicrobial resistance genes detected by WGS among 16 CRE.


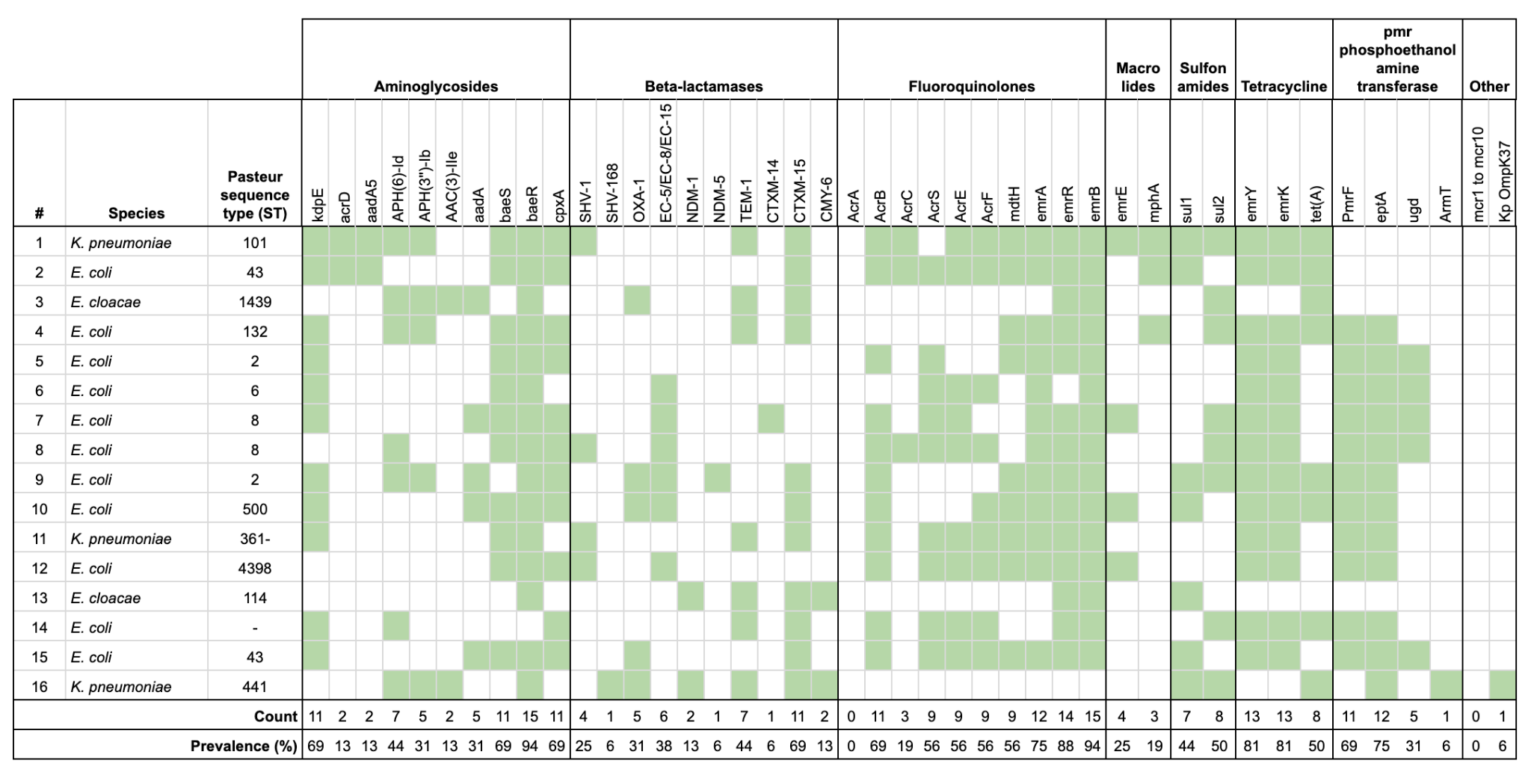


### 
